# Cystathionine β-Synthase p.S466L Mutation Causes Hyperhomocysteinemia in Mice

**DOI:** 10.1002/humu.20773

**Published:** 2008-05-02

**Authors:** Sapna Gupta, Liqun Wang, Xiang Hua, Jakub Krijt, Viktor Kožich, Warren D. Kruger

**Affiliations:** 1 Division of Population Science, Fox Chase Cancer CenterPhiladelphia, Pennsylvania; 2 Institute of Inherited Metabolic Disease, Charles University in Prague-1st Faculty of MedicinePrague, Czech Republic

**Keywords:** inborn error, metabolism, mouse model, homocystinuria, CBS

## Abstract

Missense mutations in the cystathionine β-synthase (CBS) gene are the most common cause of clinical homocystinuria in humans. The p.S466L mutation was identified in a homocystinuric patient, but enzymatic studies with recombinant protein show this mutant to be highly active. To understand how this mutation causes disease in vivo, we have created mice lacking endogenous mouse CBS and expressing either wild-type (Tg-hCBS) or p.S466L (Tg-S466L) human CBS under control of zinc inducible metallothionein promoter. In the presence of zinc, we found that the mean serum total homocysteine (tHcy) of Tg-S466L mice was 142 ± 55 µM compared to 16 ± 13 µM for hCBS mice. Tg-S466L mice also had significantly higher levels of total free homocysteine and S-adenosylhomocysteine in liver and kidney. Only 48% of Tg-S466L mice had detectable CBS protein in the liver, whereas all the Tg-hCBS animals had detectable protein. Surprisingly, CBS mRNA was significantly elevated in Tg-S466L animals compared to Tg-hCBS, implying that the reduction in p.S466L protein was occurring due to posttranscriptional mechanisms. In Tg-S466L animals with detectable liver CBS, the enzyme formed tetramers and was active, but lacked inducibility by S-adenosylmethionine (AdoMet). However, even in Tg-S466L animals that had in vitro liver CBS activity equivalent to Tg-hCBS animals there was significant elevation of serum tHcy. Our results show that p.S466L causes homocystinuria by affecting both the steady state level of CBS protein and by reducing the efficiency of the enzyme in vivo. Hum Mutat 29(8), 1048–1054, 2008. © 2008 Wiley-Liss, Inc.

## INTRODUCTION

Cystathionine β-synthase (CBS; EC 4.2.1.22; MIM # 236200) is a key enzyme in the transsulfuration pathway that regulates homocysteine homeostasis. Mutations in the *CBS* gene cause clinical CBS deficiency (homocystinuria), a metabolic disorder characterized by extreme elevations in plasma total homocysteine (tHcy). The natural history of untreated CBS deficiency includes the development of venous thrombosis, arteriosclerosis, ectopia lentis, developmental delay, and osteoporosis [Mudd et al., 2001]. The enzyme is a cytosolic homotetramer consisting of four 63-kDa subunits [Kraus et al., 1993, 1986]. CBS has three functional domains: a heme binding domain, a catalytic core domain, and a C-terminal regulatory domain [Jhee and Kruger, 2005]. The C-terminal regulatory domain binds S-adenosylmethionine (AdoMet), an allosteric effector that results in a three-fold increase in V_max_ for the enzyme [Taoka et al., 1998]. Most disease-causing mutations in CBS are of the missense variety [Kraus et al., 1999].

The p.S466L (c.1397C>T) mutation of CBS was originally described in a compound heterozygote along with a p.I278T (c.833T>C) mutation. This patient had thrombosis and elevated tHcy (167 µM) but lacked other clinical signs associated with CBS deficiency [Gaustadnes et al., 2000]. Surprisingly, expression of recombinant p.S466L in either bacterial or mammalian cellular expression systems demonstrate that p.S466L is catalytically active, although it is no longer stimulated by AdoMet [Janosik et al., 2001; Maclean et al., 2002]. Since it is known that the p.I278 T alteration is not dominant, the question arises as to why the patient had clinical disease. To address this issue we have created mouse models that lack endogenous mouse CBS and express either wild-type human CBS or p.S466L under the control of zinc inducible metallothionein promoter. Our results show that the p.S466L mutation results in reduced levels of CBS protein resulting in severe hyperhomocysteinemia in vivo.

## MATERIALS AND METHODS

### DNA and Constructs

All nucleotide numbering is based on the GenBank RefSeq NM_000071.1. The DNA mutation numbering system is based on the cDNA with +1 corresponding to the A or the ATG translation initiation codon in the reference sequence. The initiator codon is codon 1 in the protein sequence. Site-directed mutagenesis was used to introduce a c.1397C>T (p.S466L) change into pLW2, which contains a hemagglutinin epitope— tagged version of the human CBS cDNA [Wang et al., 2004] using the Quick Change XL site-directed mutagenesis kit from Stratagene (La Jolla, CA). The entire open reading frame of the resulting clone, pLW2:466, was sequenced to verify no additional changes occurred. Plasmid pLW2:466 was subsequently digested with *MfeI* and cloned into MT-LCR expression vector 2999 (obtained from Richard Palmiter, University of Washington, Seattle) [Palmiter et al., 1993]. The resulting plasmid was designated pLW3:466.

### Yeast Studies

The c.1397C>T (p.S466L) mutation was introduced into yeast strain WY35 (MATα *ura3-52 his3 trp1 cys4::LEU2)* using gap repair as previously described [Shan et al., 2001].

### Mouse Generation

Transgenic mice containing pLW3:466 were created as previously described [Wang et al., 2004]. In brief, DNA was digested with *Sal*I, purified and microinjected into the pronuclei of day 0.5 C57B6/C3 H F2 embryos. Injected embryos were then transferred to pseudopregnant mice. Tails from the resulting pups were then screened for the presence of the transgene by PCR as described [Wang et al., 2004]. DNA-positive animals were then bred to C57B6 animals and the offspring was then tested for germline transmission. Of the five pLW3:466 founders, three had germline transmission. Offspring of all three lines were given 25 mmol/L ZnSO_4_ for 10 days and examined for transgene expression by Western blot. Offspring from line EB86 gave the highest expression, so this line was chosen for further study.

To create mice that only had p.S466L CBS, transgene-positive offspring were bred to C57B6 *Cbs*^−^ heterozygous animals originally obtained from Jackson Laboratories (Bar Harbor, ME) to obtain Tg^+^ *Cbs*^−/+^ animals [Watanabe et al., 1995]. These animals were then backcrossed again to C57B6 *Cbs*^−^ heterozygous animals to obtain Tg^+^ *Cbs*^−/−^ animals. To generate large numbers of Tg^+^ *Cbs*^−/−^ animals, male Tg^+^ *Cbs*^−/−^ mice were bred with female Tg^1^ *Cbs*^−/+^. All crosses were done in the presence of 25 mmol/L ZnSO_4_ water to induce transgene expression. Tg-S466L *Cbs*^−/−^ offspring were generated at expected Mendelian frequencies and had no obvious phenotypes compared to Tg-hCBS *Cbs*^−/−^ animals.

Genotyping of offspring was generally done at the time of weaning as previously described [Wang et al., 2004]. Animals were fed standard rodent chow (Teklad 2018SX, Harlen Teklad, Madison, WI) containing 0.43% methionine by weight.

### Metabolite Measurements

Serum tHcy and methionine were measured using a Biochrom 30 amino acid analyzer (Cambridge, UK) as previously described [Wang et al., 2004]. tHcy refers to the sum total of all forms of homocysteine, including protein-bound, free-reduced, and free-oxidized. For tissue metabolites, tissues were frozen immediately after excision, ground, and deproteinized in perchloric acid to prevent possible enzymatic conversions of the metabolites. For AdoMet/S-adenosylhomocysteine (AdoHcy) analysis, we used a short (50×2.1 mm) graphite column (Hypercarb; Thermo Scientific, Waltham, MA) for retention and separation of AdoMet and AdoHcy from early eluting salts and matrix compounds. The AdoMet/AdoHcy were detected and quantified using an LCQ Advantage Max ion-trap mass spectrometer (Thermo Scientific) operated in positive-ion (ESI1) mode. Multiple-reaction monitoring conditions were optimized for AdoMet (m/z 399 →250), AdoHcy (m/z 385 →250), and stable-isotope internal standards ^2^H_3_-AdoMet (m/z 402→250) and ^13^C_5_-AdoHcy (m/z 390→255). The calibration curves were linear (r^2^>0.99) in the range 0.5–100 mmol/L, the intra- and interassay coefficients of variance (CVs) were <10%.

For tissue aminothiol analysis, the supernatant was reduced with Tris (2-carboxyethyl) phosphine (TCEP) and derivatized with ammonium7-fluorobenzo-2-oxa-1, 3-diazole-4-sulfonate (SBD-F) as described [Krijt et al., 2001]. A 10-µL aliquot of derivatized sample was injected onto an reverse phase (RP) C18 column (Prontosil C18-AQ, 250 × 4.0 mm, 3.0 µm; Bischoff Chromato-graphy, Leonberg, Germany) and aminothiols were eluted with gradient of 50 mM KH_2_PO_4_ (pH 1.9) in water to 50 mM KH_2_PO_4_ (pH 1.9) in 30% acetonitrile. The fluorescence intensities were measured with excitation at 385 nm and emission at 515 nm. Because reduction occurred after deproteinization, this procedure determines total free homocysteine, not tHcy.

### Western Blotting and CBS Enzyme Activity

Liver and yeast homogenates were prepared as previously described [Shan et al., 2001; Wang et al., 2005]. For Western blotting, 30 µg of total protein extract was electrophoresed on a 7% NuPAGE Tris-Acetate gel (Invitrogen, Carlsbad, CA) under denaturing conditions followed by transfer on to a polyvinylidene fluoride (PVDF) membrane (Bio Rad, Hercules, CA). For native Western blotting, samples were run at 4°C under nondenatured conditions. CBS was detected using a polyclonal anti-human primary antibody (1:10,000) and secondary anti-rabbit antibody (1:30,000) conjugated with horseradish peroxidase (Amersham Biosciences, Little Chalfont Buckinghamshire, UK). Signal was visualized by SuperSignal West Pico Chemiluminescent kit (Pierce, Rockford, IL) and signal was calculated using an Alpha Innotech image analyzer (San Leandro, CA).

CBS activity was analyzed in the presence and absence of AdoMet (250 µM) as previously described [Wang et al., 2004]. One unit of activity is defined as micromoles (µmol) of cystathionine formed per milligram of protein per hr. For normalization studies the amount of CBS was determined by Western analysis and the activity in the p.S466L was multiplied by the densitometry ratio between wild-type and p.S466L. For kinetic studies, a standard assay system was used, keeping one substrate at saturating conditions and varying the other from 0 to 40 mM. Data was analyzed using GraphPad Prism 4.0 software (San Diego, CA).

### Quantitative Real-Time PCR

RNA was extracted from livers using TRIzol plus RNA purification kit (Invitrogen). A total of 100 ng of RNA was used for Taq Man Gene expression analysis using two probes, one for human CBS (Hs00163925_m1; Applied Biosystems, Foster City, CA) and one for mouse β-actin (Mm00607939_s1; Applied Biosystems). Quantification of signal was achieved using an Applied Biosystems 7900 HT version 2.2.2 sequence detection system. Each sample was assayed in triplicate for both probes. Relative signal strength was calculated using the ΔΔC_t_ method.

### Statistics

All values cited in text and figures are arithmetic mean ± standard deviation (SD). *t*-Tests are all two-sided with P < 0.05 considered significant. Linear regression analysis and comparison of regression lines was performed using GraphPad Prism 4.0.

## RESULTS

### p.S466L Expressed in *S. cerevisiae* Is Constitutively Active

To determine the functional consequences of p.S466L, we expressed human p.S466L in a yeast strain in which the endogenous yeast CBS gene (*CYS4*) is deleted. As a result of this mutation this strain cannot synthesize cysteine and exhibits a cysteine auxotrophy. We found that expression of p.S466L allowed growth on cysteine-free media, indicating that the p.S466L enzyme is active (data not shown). To confirm this observation, we prepared homogenates from yeast expressing either wild-type or p.S466L and measured the amount of CBS present by Western blot and the amount of CBS activity in the absence and presence of AdoMet (Supplementary Fig. S1; available online at http://www.interscience.wiley.com/jpages/1059-7794/suppmat). We found that the amount of p.S466L protein was essentially identical to the wild-type, and that its activity in the presence of AdoMet was only slightly less than the wild-type. However, in the absence of AdoMet the p.S466L enzyme was considerably more active than the wild-type enzyme. Our data in yeast confirms previous observations that recombinant p.S466L is stable and constitutively active.

### Tg-S466L Mice Have Hyperhomocysteinemia

**FIGURE 1:**
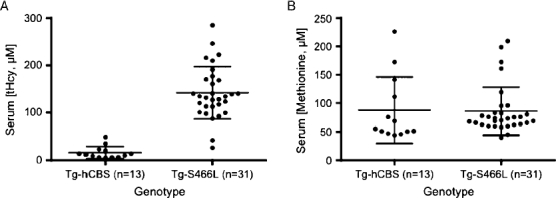
Serum tHcy and methionine inTg-S466L and Tg-hCBS mice. Mice lacking mouse CBS containing either the p.S466L or wild-type human CBS transgene were induced with zinc water for **10** days. Serum was then collected and analyzed for tHcy and methionine. A: Distribution of tHcy measurements. Dots indicate individual animals. Lines indicate average and standard deviation. The difference between the distributions is highly significant (P < 0.0001). **B**: Distribution of serum methionine concentrations.

To determine the effect of p.S466L mutation on serum tHcy, we created two genetically engineered mouse strains. These mice lack endogenous mouse CBS and contain a transgene that expresses either the wild-type human CBS protein (Tg-hCBS; see Wang et al. [2004]) or the p.S466L protein (Tg-S466L, see Materials and Methods) from a mouse metallothionein promoter. This promoter can be activated in the liver and kidney by the addition of zinc to the drinking water. Adult mice were kept on zinc water for 10 days and then analyzed for their tHcy. The Tg-hCBS mice (n = 13) had an average tHcy of 16±13 µM. In contrast, the Tg-S466L mice (n = 31) had an average tHcy of 142±55 µM (hCBS vs. p.S466L; P < 0.0001; Mann-Whitney U-test) (Fig. 1A). Interestingly, these animals exhibited wide variation in their tHcy, ranging from 26 to 285 µM. Since human patients with hyperhomocysteinemia tend to have hypermethionemia as well, we also measured serum methionine levels [Mudd et al., 2001]. We did not observe a statistically significant difference in serum methionine (Fig. 1B). These results indicate that expression of p.S466L in mice causes hyperhomocysteinemia with an absence of hypermethionemia.

**FIGURE 2:**
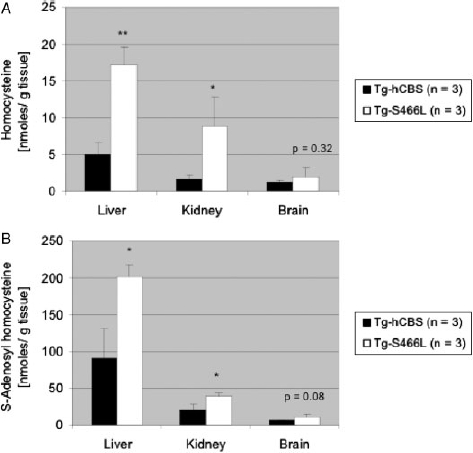
Total free homocysteine and AdoHcy levels in tissues. Total free homocysteine and AdoHcy were measured in liver, kidney, and brain from Tg-hCBS and Tg-S466L animals as described in Materials and Methods. **A**:Tissue homocysteine content. Mean and standard deviation are shown. **B**:Tissue AdoHcy content. **P < 0.005; *P < 0.05.

We also measured the total free concentration of several homocysteine-related metabolites in the liver, kidney, and brain of Tg-hCBS and Tg-S466L mice. These metabolites included cysteine, homocysteine, glutathione, cysteinyl-glycine, gamma-glutamylcysteine, AdoMet, and AdoHcy. In the liver and kidney, Tg-S466L mice had large elevations of both total free homocysteine and AdoHcy (Fig. 2) as would be expected in CBS deficiency. We did not observe any significant differences in any of the other metabolites examined.

### Variable Expression of p.S466L Protein

**FIGURE 3:**
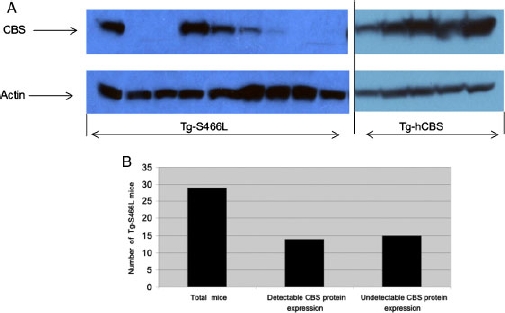
CBS protein levels in the liver. Mice carrying the indicated transgene were induced with zinc water for 10 days and liver extracts were assessed for CBS by Western blot. **A**: Representative Western blots showing the variable expression of CBS in Tg-S466L mice and in comparison with Tg-hCBS mice. Actin loading control is shown below. **B**: Bar graph summarizing Western data for all 29 Tg-S466 mice assessed for liver CBS protein.

Although most of the Tg-S466L mice exhibited the severe hyperhomocysteinemia (of more than 100 µM), the high degree of variability in their tHcy led us to examine the steady state levels of p.S466L protein in mouse liver. Western blot analysis indicated that there was large variation in the steady state levels of p.S466L protein (Fig. 3A). Of the 29 p.S466L mice examined for CBS protein levels, 15 (52%) showed no detectable CBS protein, while the rest generally had significantly less CBS than observed in control Tg-hCBS mice (Fig. 3B). Animals with detectable CBS activity had slightly lower mean tHcy compared to nondetectable animals, but the difference was not statistically significant (128 vs. 137 µM; P = 0.59). However, it should be noted that 5 of the 6 animals with tHcy under 100 µM had detectable CBS levels (data not shown).

### Elevated p.S466L mRNA inTg-S466L Mice

To understand the reason for variability and low-level expression of p.S466L CBS, we measured steady state levels of human CBS mRNA in the livers of Tg-hCBS and Tg-S466L mice. We found that all of the p.S466L animals had significantly more CBS mRNA compared to the Tg-hCBS animals (Supplementary Fig. S2). The animals lacking CBS expression had on average 5.5-fold more CBS mRNA, while the animals with detectable expression had on average 10.2-fold more CBS mRNA. These findings show that there is no problem with induction of the transgene by zinc in Tg-S466L mice and implies that the effect of p.S466L mutation is to reduce either protein production or stability.

### Tg-S466L Is EnzymaticallyActive, FormsTetramers, and Lacks AdoMet Inducibility

**FIGURE 4:**
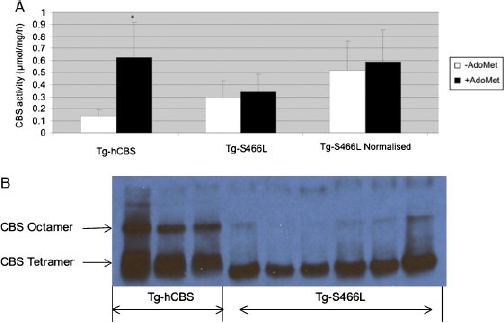
Examination of p.S466L and wild-type CBS enzyme activity and multimers formation. **A**: Enzyme activity and AdoMet response of wild-type and p.S466L CBS. The first set of columns shows CBS activity in liver extracts derived from Tg-hCBS mice in the absence and presence of AdoMet (n = 3). The second set of columns shows the same for Tg-S466L mice (n = 6). The third set shows the relative Tg-S466L activity when adjusted for total p.S466L protein as judged by Western blot analysis. **B**: Comparison of the CBS protein in Tg-hCBS and expressing Tg-S466L mice livers using native gels followed by Western blot analysis.

To further characterize the behavior of p.S466L CBS, we measured CBS enzyme activity and tetramer formation in the liver homogenates of Tg-hCBS and Tg-S466L animals with detectable CBS levels. First, we assessed CBS activity in the presence and absence of allosteric activator AdoMet (Fig. 4A). We found that CBS activity in Tg-hCBS liver homogenates (n = 3) was 0.143±0.05 µmol/mg/hr in the absence of AdoMet and increased to 0.63 ± 0.29 µmol/mg/hr, (∼4-fold; P < 0.05; *t*-test) in the presence of 250 µM of AdoMet. In contrast, we found that Tg-S466L liver homogenates (n = 6) with detectable CBS protein expression had an insignificant change in CBS activity, from 0.30 ± 0.14 µmol/mg/hr in the absence of AdoMet to 0.33 ± 0.15 µmol/mg/hr in the presence of AdoMet. When the enzyme activity of the Tg-S466L mice was normalized for CBS protein levels, the specific activity of p.S466L was found to be almost equivalent (0.6 ± 0.26 µmol/mg/hr) to wild-type CBS in the presence of AdoMet. These findings are consistent with the yeast and *E. coli* studies indicating that p.S466L results in a constitutively active CBS protein [Janosik et al., 2001; Maclean et al., 2002].

We also examined the effect of the p.S466L mutation on tetramer formation by utilizing native gels followed by Western blotting (Fig. 4B). Again, we focused on Tg-S466L animals that had detectable liver CBS protein. Our results show that the p.S466L mutation does not cause a significant defect in tetramer formation of the protein, although there may be a slight reduction in the amount of higher-order octamer observed. When the enzyme activity of the expressing Tg-S466L mice was normalized for CBS tetramer levels, specific activity of p.S466L was again found to be almost equivalent (0.53 ± 0.23 µmol/mg/hr) to wild-type hCBS in the presence of AdoMet (data not shown). This data indicate that the p.S466L protein that is present in mouse liver is enzymatically active, forms tetramers, but lacks AdoMet inducibility.

### Relationship Between Liver CBS Protein, Activity, and tHcy

**FIGURE 5:**
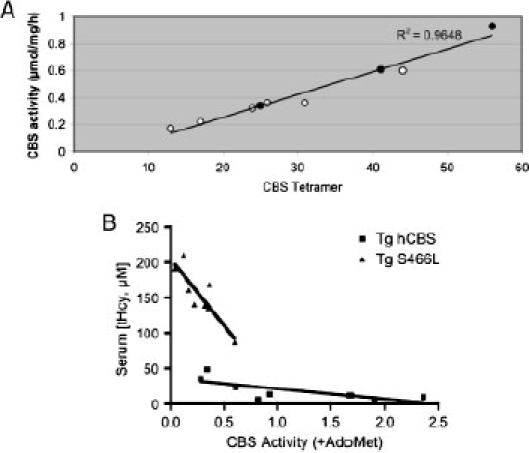
Correlation between CBS activity, tetramer formation, and serum tHcy. **A**: Correlation between CBS tetramer density and CBS activity (in presence of AdoMet) in the livers of mice of respective genotype. Black dots represent Tg-hCBS mice and white dots represent Tg-S466L mice. **B**: Correlation between liver CBS activity (in presence of AdoMet) and serum tHcy in Tg-hCBS (n = 8) and Tg-S466L (n = 9) mice. CBS activity has been measured in the terms of micromoles (µmol) of cystathionine formed per milligram of protein per hr.

We examined the relationship between the amount of CBS tetramer present in liver homogenates from Tg-S466L and Tg-hCBS animals and CBS activity. We found that there was a strong linear relationship between the level of tetramer and CBS activity for both Tg-hCBS and Tg-S466L animals (R^2^ = 0.96; Fig. 5A). Both the wild-type and the p.S466L animals appear to lie along the same line. From these findings we conclude that the amount of tetramer formed is a good predictor of CBS activity.

We also examined the relationship between liver CBS activity and serum tHcy from mice with detectable liver CBS. We found that there was a linear relationship for both Tg-hCBS and Tg-S466L mice, but that the slope of the regression line differed dramatically between the two groups (P < 0.0003; Fig. 5B). For the Tg-hCBS animals each one-unit decrease in liver CBS activity was associated with a 14-µM increase in tHcy, while for Tg-S466L a one-unit decrease in activity was associated with a 188-µM increase. Furthermore, there were five p.S466L animals with unnormalized liver CBS enzyme activity higher than the lowest of the Tg-hCBS animals, but they still had substantially higher serum tHcy. These findings suggest that in vivo p.S466L CBS is less effective than wild-type CBS at lowering tHcy levels.

### p.S466L Does Not Affect K_n_ or Serine

One possible explanation for the reduced effectiveness of p.S466L in lowering tHcy would be that p.S466L might affect the binding affinity (K_m_) of the enzyme. To examine this, we determined the steady state kinetic parameters of p.S466L and wild-type CBS present in mouse liver extracts. For this analysis, we specifically chose two mice that had nearly identical CBS protein levels as judged by Western blot analysis, but had very different tHcy. As shown in Table 1, both wild-type and p.S466L CBS had nearly identical K_m_ values for serine, while the p.S466L enzyme had a slightly lower K_m_ value for homocysteine. These results show that the p.S466L mutation does not adversely affect substrate binding, and that this could not be the explanation for the elevated tHcy observed in p.S466L animals.

**Table 1:** Characterization of Wild-Type and S466L Enzyme From Liver

		Homocysteine (+AdoMet)^a^		L-Serine (+AdoMet)^b^	
			
Genotype	tHcy [µM]K	K_m_ (mM)	V_max_ (µmol/mg/hr)K	K_m_ (mM)	V_max_ (µmol/mg/hr)
Tg-hCBS	48	2.5 ± 0.23	0.63 ± 0.02	0.78 ± 0.10	0.54 ± 0.01
Tg-S466L	168	1.1 ± 0.05	0.60 ± 0.01	0.77 ± 0.09	0.56 ± 0.01

a L-Serine was used at 10 mM saturating concentration.

b DL-Homocysteine was used at a saturating concentration of 20 mM.

## DISCUSSION

The experiments in this work were motivated by the apparent paradox presented by the p.S466L mutation: How can a CBS enzyme that is enzymatically active in vitro cause homocystinuria in vivo? In the present study, we found that mice expressing p.S466L exhibited hyperhomocysteinemia, confirming that this mutation is disease causing in vivo. The reduced function in vivo appears to be caused by two processes: 1) the steady state level of p.S466L protein is generally reduced compared to wild-type human CBS; and 2) even when the p.S466L protein is present, it is not as efficient in lowering plasma homocysteine levels in vivo.

As shown in Fig. 1, we observed a high level of variability in the tHcy of our Tg-S466L animals. Part of the explanation for this variability is explained by variable CBS expression in the livers of these mice. Over half of the Tg-S466L mice had no detectable CBS expression in the liver and the expressing ones generally had lower CBS levels compared to the control Tg-hCBS mice. The reduced expression of Tg-S466L protein was not caused by a reduction in the amount of CBS mRNA produced from the mutant transgene construct. In fact, the p.S466L mice had 5 to 10-fold more CBS mRNA. These findings show that the reduced levels of p.S466L protein observed in the mice is due to posttranscriptional mechanisms. Some possible mechanisms could include reduced translational efficiency of the message or increased rates of protein misfolding and subsequent proteolysis. We think it is unlikely that reduced translational efficiency is the explanation as the leucine encoded by the mutant TTG codon has a higher frequency of usage in the human genome than the wild-type TCG codon [Guigo, 1999]. Therefore, we favor the idea that in mammalian liver only a small proportion of the p.S466L protein folds correctly into a native conformation, and that the misfolded protein is subsequently degraded.

Previous studies have found that recombinant p.S466L expressed in either *E. coli* or Chinese hamster fibroblasts is stable and has enzymatic activity, but that this activity is nonresponsive to stimulation by AdoMet [Janosik et al., 2001; Maclean et al., 2002]. In both *E. coli* and Chinese hamster fibroblasts the V_max_ for the enzyme was estimated to be similar to that of the wild-type CBS enzyme assayed in the presence of AdoMet, implying that the enzyme is constitutively activated. We found essentially identical results in the characterization of p.S466L produced either in *S. cerevisiae* or in the livers of Tg-S466L mice. We failed to see induction of activity by AdoMet and the normalized activity of the mutant enzyme in vitro was essentially equivalent to the activity observed in Tg-hCBS mice. We also observed that the p.S466L enzyme that was present in the liver formed tetramers efficiently, and that the amount of tetramer present in extracts was an excellent predictor of in vitro enzyme activity. These findings confirm that when p.S466L protein is present, it is active and forms functional tetramers.

Besides affecting the amount of steady-state CBS protein, our results also suggest that the p.S466L enzyme is less efficient at lowering serum tHcy. This is shown by the relationship between liver CBS activity (measured in the presence of AdoMet) and tHcy in Tg-S466L and Tg-hCBS animals (Fig. 5B). In both Tg-hCBS and Tg-S466L mice, there was an inverse linear relationship between CBS activity and tHcy but the slope and intercepts of the lines were significantly different. One possible explanation for the difference in behavior would be if the p.S466L mutation affected substrate binding (K_m_) such that more enzyme would be required to metabolize homocysteine. However, our kinetic studies do not reveal any significant difference in K_m_ that would explain this result (Table 1). We can think of three possible hypotheses to explain these findings. First, it may be that there is some as yet unidentified allosteric effector that stimulates CBS activity in vivo, and that p.S466L does not respond to this effector. Second, mammalian CBS is known to bind heme and there is evidence that the enzyme is 50% less active under strong reducing conditions in vitro [Taoka et al., 1998]. It is possible that the p.S466L mutation may be much more severely impaired to the reducing conditions found inside the cell and that this is not observed in the standard in vitro assay conditions used in this study. A final possibility is that the p.S466L mutant form of the enzyme may be sequestered inside the cell in such a way that it is nonfunctional, but when extracts are prepared this sequestration is somehow eliminated. One possible candidate for the sequestering molecules would be molecular chaperone proteins such as Hsp70, which are known to bind to misfolded cytoplasmic proteins, and either helps them refold or directs their degradation via the carboxy terminus of Hsc70 interacting protein (CHIP) protein [McDonough and Patterson, 2003].

The findings concerning p.S466L suggest that caution must be used when examining the functional aspects of missense mutations using recombinant proteins. In general when missense mutations are identified, their functional effects are tested on recombinant proteins produced either in *E. coli* or mammalian cell culture systems. Our results demonstrate that missense alterations may have subtle effects on protein stability that vary depending on the expression system that is used. The ability to test functional effects of human mutations in the mouse probably represents the closest approximation to the situation in humans.
